# Peptide microarray profiling identifies phospholipase C gamma 1 (PLC-γ1) as a potential target for t(8;21) AML

**DOI:** 10.18632/oncotarget.18631

**Published:** 2017-06-27

**Authors:** Hasan Mahmud, Frank J.G. Scherpen, Tiny Meeuwsen de Boer, Harm-Jan Lourens, Caroline Schoenherr, Matthias Eder, Michaela Scherr, Victor Guryev, Eveline S. De Bont

**Affiliations:** ^1^ Department of Pediatric Oncology/Hematology, Beatrix Children’s Hospital, University Medical Center Groningen, University of Groningen, Groningen, The Netherlands; ^2^ Stephenson Cancer Center, The University of Oklahoma Health Sciences Center, Oklahoma City, OK, USA; ^3^ Department of Hematology, Hemostasis, Oncology and Stem Cell Transplantation, Hannover Medical School, Hannover, Germany; ^4^ Laboratory of Genome Structure and Aging, European Research Institute for the Biology of Aging, University Medical Center Groningen, University of Groningen, Groningen, The Netherlands

**Keywords:** AML, leukemia, t(8;21), PLC-γ1, peptide microarray

## Abstract

The t(8;21) (q22;q22) chromosomal translocation is one of the most frequent genetic alterations in acute myeloid leukemia (AML) which has a need for improved therapeutic strategies. We found PLC-γ1 as one of the highest phosphorylated peptides in t(8;21) AML samples compared to NBM or CN-AML in our previous peptide microarray. PLC-γ1 is known to play a role in cancer progression, however, the impact of PLC-γ1 in AML is currently unknown. Therefore, we aimed to study the functional role of PLC-γ1 by investigating the cellular growth, survival and its underlying mechanism in t(8;21) AML.

In this study, PLC-γ1 expression was significantly higher in t(8;21) AML compared to other karyotypes. The PLC-γ1 protein expression was suppressed in AML1-ETO knock down cells indicating that it might induce kasumi-1 cell death. ShRNA-mediated PLC-γ1 knockdown in kasumi-1 cells significantly blocked cell growth, induced apoptosis and cell cycle arrest which was explained by the increased activation of apoptotic related and cell cycle regulatory protein expressions. Gene expression array analysis showed the up-regulation of apoptotic and DNA damage response genes together with the downregulation of cell growth, proliferation and differentiation genes in the PLC-γ1 suppressed kasumi-1 cells, consistent with the observed phenotypic effects. Importantly, PLC-γ1 suppressed kasumi-1 cells showed higher chemosensitivity to the chemotherapeutic drug treatments and lower cell proliferation upon hypoxic stress.

Taken together, these *in vitro* finding strongly support an important role for PLC-γ1 in the survival of t(8;21) AML mimicking kasumi-1 cells and identify PLC-γ1 as a potential therapeutic target for t(8;21) AML treatment.

## INTRODUCTION

Acute myeloid leukemia (AML) forms a spectrum of diseases that share clinical and pathological features, which arise from a wide diversity of abnormalities, including mutations, cytogenetic abnormalities, and epigenetic changes [1–4]. The t(8;21)(q22;q22) rearrangement considers the most common chromosomal translocation in AML and consists of a transcript encoding for the fusion protein AML1-ETO (A-E or RUNX1-RUNX1T1) [5]. AML1–ETO is a critical step for the pathogenesis of this type of myeloid leukemia, however, it requires one or more additional mutations to cause leukemia [6–9]. The role of AML1–ETO in the t(8;21) translocation positive AML was previously studied and it appeared as a promising target, although the effectiveness of this therapy remains unclear [10–12]. Patients diagnosed with t(8;21) AML undergo conventional intensive chemotherapy and have a relatively favorable prognosis compared with other types of AMLs [13]. Despite the great clinical improvements that have been made in the treatment of AML, t(8;21) AML remains a significant clinical problem for both children and adults. The relapse rate of t(8;21) AML in children is about 30% and long-term survival rate is about 75% which indicate the need for improved therapeutic strategies [14–18]. Although molecular analysis such as next-generation sequencing and transcriptome analysis have revealed many previously unknown recurrent mutations and additional mechanistic insights such as alternations in the epigenetic status, still the results as well as the prediction of drug response to treatments with specific newly designed inhibitors in AML are missing. Single kinase inhibitor therapy can default when cancer cells bypass through alternative routes, adaptation in redundancy, and escape mechanism [19–20]. Deregulated protein phosphorylation by aberrant protein kinase activity is frequently observed in cancer, e.g. leukemia [20]. Aberrant expression of multiple signal transduction pathways is associated with a worse prognosis [21]. In order to circumvent the constraints of a certain kinase inhibitor, it is desirable to elucidate signaling networks to identify suitable targets that are essential in leukemic cell proliferation and survival to develop the most successful combination therapy approach for AML-specific subgroups. Therefore, we performed peptide microarray profiling array as we did previously [20, 22] for t(8;21) AML and found that phospholipase C-gamma 1 (PLC-γ1) at tyrosine 783 was highly phosphorylated in these leukemia cells.

PLC-γ1 can be phosphorylated at tyrosine residues (Y783, Y771) and also at a serine residue (S1248). Among these residues, PLC-γ1_Y783 is known to be a critical phosphorylation site for PLC-γ1 enzymatic activation whereas PLC-γ1_S1248 negatively regulates PLC-γ1 activity [23, 24]. PLC-γ1 is a key signaling molecule that hydrolyzes phosphatidylinositol-4,5-biophosphate to generate inositol-1,4,5-triophosphate (IP3) and 1,2-diacylglycerol (DAG), which in turn, activate intracellular Ca^2+^ and protein kinase C (PKC) signaling pathways [25]. PLC-γ1 is activated by binding to tyrosine kinases and subsequent phosphorylation and transduces signals to the downstream PKC–RAF–MEK–MAPK pathway. These pathways are known for their enhancement of cell proliferation, cell migration and cell survival when activated. Specifically, PLC-γ1 plays a critical role in vascular endothelial growth factor (VEGF) mediating signaling in endothelial cells. Interestingly, aberrant VEGF signaling promotes autocrine AML blast cell proliferation, survival, and chemotherapy resistance [26]. PLC-γ1 activity is regulated by PI3K through the interaction of the PI3K product PIP3, and PLC-γ1 PH domain. The role of PLC-γ1 in cancer progression specifically in carcinomas were extensively studied [27–29]. Its role, if any, in AML progression is not studied yet. Markova B, et al. underscored the possible importance of PLC-γ1 in chronic myeloid leukemia (CML) leukemogenesis via a novel Akt-independent, PLC-γ1-driven mechanism of activation of mTOR/p70S6-K in BCR-ABL-positive cells [30]. Together, further studies to investigate the role of PLC-γ1 in other types of leukemia are necessary.

In this study, we aimed to study the functional role of PLC-γ1 in t(8;21) AML and its interference with signaling networks. Briefly, we identified the higher PLC-γ1 expression in t(8;21) AML patients and we studied the effects of loss of PLC-γ1 in kasumi-1 and gene expression profiles using shRNA-mediated suppression. In addition, the chemosensitivity of PLC-γ1 suppressed cells was evaluated.

## RESULTS

### Peptide microarray profiling identifies PLC-γ1 as a potentially druggable target for t(8;21) leukemia

**Figure 1:**
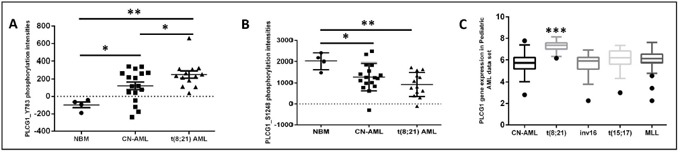
Higher PLC-γ1 peptide phosphorylation in t(8;21) AML Dot plot of mean peptide intensity of PLC-γ1 peptides were compared between t(8;21) AML (n=13) compared to CN-AML (n=17) or NBM (n=4). **(A)** Phosphorylation of PLC-γ1 at tyrosine residue 783 was significantly highly phosphorylated in t(8;21) AML compared to CN-AML or NBM. **(B)** Phosphorylation of PLC-γ1 at serine residue 1248 was significantly lowly phosphorylated in t(8;21) AML compared to CN-AML or NBM. **(C)** Gene expression of PLC-γ1 among different AML karyotypes from a publicly available pediatric AML gene array data set, showed that PLC-γ1 was significantly highly expressed in t(8;21) AML.

Phospholipase C-gamma 1 (PLC-γ1_Y783) was identified from our previous peptide array (unpublished data) as one of the highest phosphorylated peptides in t(8;21) AML samples when compared to NBM or CN-AML (Figure 1A). It is known that phosphorylation of PLC-γ1 at Y783 increases the PLC-γ1 enzymatic activity and PLC-γ1 phosphorylation at S1248 inhibits the PLC-γ1 enzymatic activity. From our data, it could be appreciated that phosphorylation status of PLC-γ1_Y783 was vice versa to the phosphorylation of PLC-γ1_S1248 in t(8;21) AML (p<0.05 and p<0.01, Figure 1A and p<0.05, p<0.01, Figure 1B). About 38% (5/13) t(8;21) samples had c-KIT mutations and no significant difference of PLC-γ1_Y783 phosphorylation level was found between the t(8;21) samples with or without KIT mutation (data not shown). The expression profile of PLC-γ1 in a publicly available pediatric AML database (http://r2.amc.nl) also showed significant highest expression of PLC-γ1 in t(8;21) AML when compared to other AML karyotypes (p<0.001, Figure 1C). Since the role of PLC-γ1 is currently unknown in t(8;21) AML, we aim to study the functional role of PLC-γ1 in t(8;21) AML using kasumi-1 cell line by studying its effect on cellular growth and survival and underline mechanisms.

### PLC-γ1 is highly expressed in primary t(8;21) AML samples and AML cell lines. Downregulation of core RUNX1/ETO (AML1-ETO) in kasumi-1 cells reduced PLC-γ1 expression

**Figure 2:**
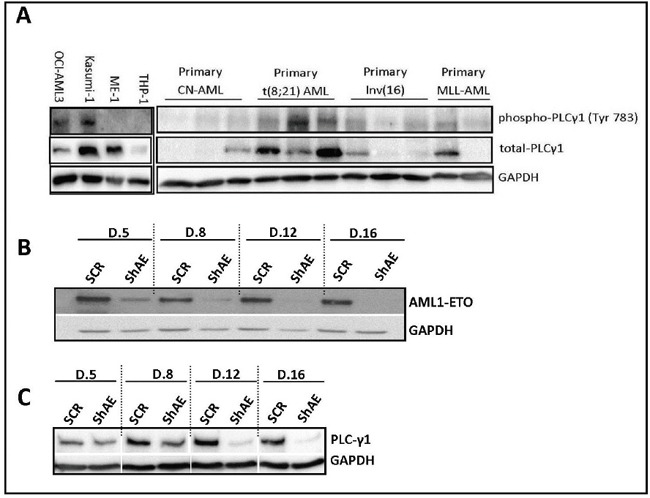
PLC-γ1 expression in AML cell lines, primary AML samples, and AML1-ETO suppressed kaumi-1 cells **(A)** Western blot analysis confirms the higher PLC-γ1 expression in kasumi-1 cells compared to other cell lines (OCI-AML3, ME-1 and THP-1). Similarly, higher PLC-γ1 expression (both phospho-PLC-γ1 at Y783 and total PLC-γ1 protein) was found in t(8;21) AML primary samples (n=3) compared to other primary AML (CN-AML, inv16 and MLL-rearranged AML). **(B)** shRNA of AML1-ETO (RUNX1/ETO) in kasumi-1 cells, confirmed the AML1-ETO suppression in a day dependent manner by Western blot analysis. **(C)** Downregulation of PLC-γ1 protein was observed in suppressed AML1-ETO kasumi-1 cells in a day dependent manner by western blot analysis

We checked the protein expression PLC-γ1 (both phospho and total PLC-γ1) by western blot analysis in different AML cell lines and primary AML samples to confirm PLC-γ1 peptide phosphorylation data from the peptide microarray (Figure 2A). PLC-γ1 expression of both total-PLC-γ1 and phospho-PLC-γ1 were found in AML primary samples among them t(8;21) AML samples showed the highest protein expression. Similarly, kasumi-1 cells and t(8;21) AML showed the highest level of phospho-PLC-γ1 and total-PLC-γ1 expression among others. Furthermore, we used the *RNA* interference approach of AML1-ETO (*shAE*) for the elucidation of the dependency of PLC-γ1 expression in the t(8;21)-positive AML kasumi-1 cell line (Figure 2B, 2C). Figure 2B showed clearly less AML1-ETO protein expression compared to control plasmid. The expression of PLC-γ1 was reduced overtime after transduction with shAE compared with control (Figure 2C). Furthermore, we checked if the consensus sequence of AML1-ETO (TGTGGT) could be found in any region of the PLC-γ1 gene by sequence matching. AML1-ETO consensus sequence is present in (−) 4081 base pairs upstream of transcription start site (TSS) of the PLC-γ1 which could be a regulatory region of PLC-γ1 (data not shown).

### Suppression of PLC-γ1 in kasumi-1 cells inhibit cell growth, induced apoptosis and cell cycle arrest

**Figure 3:**
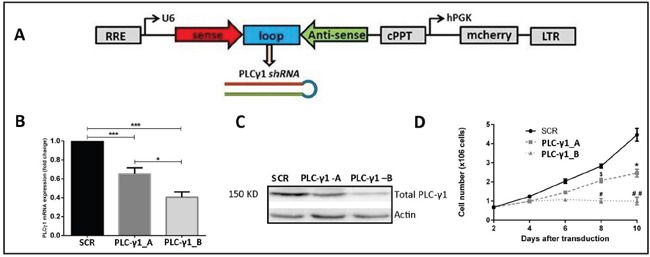
PLC-γ1 is essential for kasumi-1 cell growth **(A)** Schematic diagram for generating the shRNA construct for PLC-γ1. **(B)** Two shRNAs of PLC-γ1 were used (named as; PLC-γ1-A and PLC-γ1-B). PLC-γ1 was successfully downregulated in kasumi-1 cells which was confirmed by RT-PCR. **(C)** Quantification of PLC-γ1 at the protein level in transduced kasumi-1 cells by western blot confirming the PLC-γ1 downregulation. **(D)** Growth curve analysis shows that PLC-γ1 downregulation results in a decrease cell growth in kasumi-1 cells (n=4). * denoted the comparison between SCR vs PLC-γ1_A; # denoted the comparison between SCR vs PLC-γ1_B and $ denoted the comparison between PLC-γ1_A vs PLC-γ1_B.

The design of *shPLC-γ1*construct is shown in the Figure 3A. Two different lentiviral vectors were used: *shPLC-γ1* that targeted the PLC-γ1 mRNA; and shSCR encoded for a nonspecific scrambled (SCR) shRNA. Two *shPLC-γ1* constructs (PLC-γ1-A and PLC-γ1-B) were prepared for the transduction. The *shPLC-γ1* expressing cells showed ∼35% (PLC-γ1-A) and ∼60% (PLC-γ1-B) decrease in PLC-γ1 mRNA level compared with the control (p<0.05 and P<0.001, Figure 3B). These results were confirmed by PLC-γ1 protein level analysis by western blotting (Figure 3C). The shRNA-mediated silencing of PLC-γ1 leads to significant suppression of the kasumi-1 cell growth after day 8 of transduction (p<0.05, Figure 3D).

### Downregulation of PLC-γ1 in kasumi-1 cells induced apoptosis and cell cycle arrest

**Figure 4:**
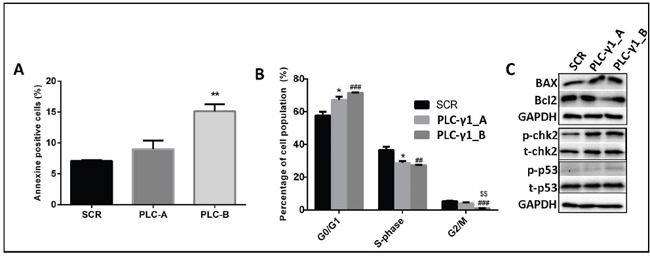
PLC-γ1 downregulation induces apoptosis and cell cycle arrest in kasumi-1 cells **(A)** Percentage of apoptosis (Annexin V-positive) at day 4 after transduction (n=4) shows an increase apoptotic cell number for PLC-γ1 downregulated cells (PLC-γ1-B construct only) as compared to control transduced cells. **(B)** Cell cycle analysis shows a reduction in the percentage of cells in S and G2/M phases with a concomitant increase of cells in G0/G1 phases in PLC-γ1 downregulated cells in comparison with control shRNA (SCR). * denoted the comparison between SCR vs PLC-γ1_A; # denoted the comparison between SCR vs PLC-γ1_B and $ denoted the comparison between PLC-γ1_A vs PLC-γ1_B. **(C)** Immunoblot analysis confirms the significant increase of pro-apoptotic BAX protein and phosphorylation of chk2 protein in PLC-γ1 downregulated cells compared to SCR.

To elucidate the nature of the cell growth suppression, we measured an impact of PLC-γ1 downregulation on the apoptosis. The percentage of Annexin V-positive kasumi-1 cells of *PLC-γ1-B* transduced cells was significantly higher than in *shSCR*-expressing cells (p<0.01, Figure 4A). Thus, the proof of principal results strongly supports that PLC-γ1 might be important in t(8;21) AML survival as suppression of PLC-γ1 inhibited cell growth and induced apoptosis in kasumi-1 cells. To explain the dramatic effect of PLC-γ1 knockdown on cell growth, we studied the consequences of PLC-γ1 knockdown on cell cycle progression. We hypothesized that the phenotypic effects of growth inhibition and the induction of apoptosis could be assigned to a defect in cell cycle regulation. Cell cycle analysis of PLC-γ1 knockdown cells revealed a significant increase of the percentage of cells in G0/G1 phase (p<0.05 and p<0.001) of cell cycle, significantly decrease in S-phase and G2/M (p<0.05, p<0.01 and p<0.001) suggesting G0/G1 phase cell cycle arrest (Figure 4B). The exact changes of cell cycle phases are also shown with the original dot plot from the FACS analysis (Supplementary Figure 1). To understand the signal transduction that could explain PLC-γ1 knockdown mediated cell death in kasumi-1 cell, we analyzed proteins involved in apoptosis and cell cycle regulation. Immunoblot analysis showed an induction of pro-apoptotic BAXa/b and a slight downregulation of anti-apoptotic Bcl-2 in PLC-γ1 transduced cells (Figure 4C). Simultaneously, to gain more insights into the observed cell-cycle regulation, we found significant increase in p-Chk2 by PLC-γ1 knockdown of kasumi-1 cells (Figure 4C). The quantification of the western blot analysis is presented as Supplementary Figure 2. These data implicates that PLC-γ1 knockdown may induce apoptosis and increase G0/G1 cell-cycle arrest with the decrease S-phase and G2/M phase in kasumi-1 cells by enhancing p53 DNA binding that prevents the AML cells from entering mitosis and finally results in apoptosis.

### Gene expression profiling of PLC-γ1 knockdown cells

To understand the changes of transcriptional consequences of the *PLC-γ1* knockdown in kasumi-1 cell, we performed the gene expression microarray profiling; using the transduced kasumi-1 cells of *SCR*, *shPLC-γ1-A* and *shPLC-γ1-B*. Since we observed strong phenotypes of growth inhibition, apoptosis induction and cell cycle arrest upon PLC-γ1 knockdown, therefore, we made an overview of interesting genes that were either up- or downregulated in *PLC-γ1* (Table 1). The mRNA microarray data confirmed that a list of genes related to apoptosis (*BAX*, *BCL2L12, PRKCD, STAT3, CASP8, APAF1)* and DNA damage response (*ATM*, *RAD9, BARD1, XRCC5, EXO1, RBL2, ERBB3, MAPK14*) were upregulated in *shPLC-γ1* samples whereas genes related to cell growth (*BID*, *IKBKB, NFKB1*), proliferation or differentiation (*eIF2S1, CDKN2B, PKCH, GFAP, ID1, PRDM1, p120GAP, ARHGEF10*) were downregulated in this *shPLC-γ1* samples. Interestingly, we observed downregulation of two important calcium signaling regulatory genes CAMK2B and RYR1 which are known to be downstream of PLC-γ1 signaling.

**Table 1:** List of up- and downregulated genes in both *shPLC-γ1-A* and *shPLC-γ1-B* versus *shSCR* transduced cells

*Upregulated genes in PLC-γ1 KD cells*	*Down regulated genes in PLC-γ1 KD cells*
***Apoptosis related genes***	***Genes involved in Cell growth and survival***
***BAX*** *(BCL2-associated X protein)*	***BID*** *(BH3 interacting domain death agonist)*
***BCL2L12*** *(BCL2 Like 1)*	***IKBKB****(Inhibitor of Kappa light polypeptide gene enhanceriIn B-Cells, kinase Beta)*
***PRKCD*** *(Protein Kinase C Delta)*	***NFKB1****(Nuclear Factor Kappa B Subunit 1)*
***STAT3****(Signal Transducer And Activator Of Transcription 3)*	***Genes involved in Cell proliferation and differentiation***:
***CASP8****(Caspase 8)*	***eIF2S1****(Eukaryotic Translation Initiation Factor 2 Subunit Alpha)*
***APAF1*** *(Apoptotic protease activating factor 1)*	***CDKN2B****(Cyclin Dependent Kinase Inhibitor 2B)*
***DNA damage and cell cycle regulated genes***	***PKCH****(Protein Kinase C Eta)*
***ATM****(Ataxia Telangiectasia Mutated)*	***GFAP*** *Glial Fibrillary Acidic Protein (Glial Fibrillary Acidic Protein)*
***RAD9****(RAD9 Checkpoint Clamp Component B)*	***ID1*** *(Inhibitor Of DNA Binding 1, HLH Protein)*
***BARD1****(BRCA1 Associated RING Domain 1)*	***PRDM1****(PR/SET Domain 1)*
***XRCC5****(X-Ray Repair Cross Complementing 5)*	***p120GAP*** *(RAS P21 Protein Activator 1)*
***EXO1****(Exonuclease 1)*	***ARHGEF10*** *(Rho Guanine Nucleotide Exchange Factor 10)*
***RBL2****(RB Transcriptional Corepressor Like 2)*	***Genes involved in Calcium signaling***:
***ERBB3****(Erb-B2 Receptor Tyrosine Kinase 3)*	***CAMK2B*** *(Calcium/Calmodulin Dependent Protein Kinase II Beta*)
***MAPK14****(Mitogen-Activated Protein Kinase 14)*	***RYR1*** *(Ryanodine receptor 1)*

### Functional importance of PLC-γ1 in kasumi-1 cells under stress conditions (chemotherapeutic drugs and hypoxia)

**Figure 5:**
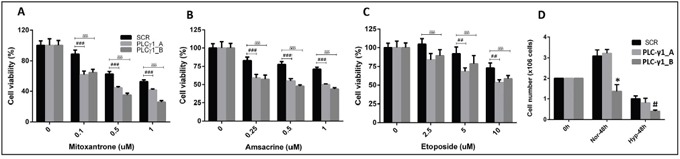
Functional importance of PLC-γ1 in kasumi-1 cells under stress conditions (chemotherapeutic drugs and hypoxia) **(A-C)** PLC-γ1 knockdown cells at day 4 after transduction were shown to significantly induce cellular sensitivity to the genotoxic agents; mitoxantrone (p<0.05, p<0.001), amsacrine (p<0.01, p<0.001) and etoposide (p<0.05, p<0.01 and p<0.001) in kasumi-1 in a dose-dependent manner. **(D)** Cell viability was found to be reduced in all three conditions upon 48h hypoxic condition (Hyp-48h). Kasumi cells with PLC-γ1_B shRNA was significantly reduced (p<0.05) compared to scramble (SCR). * denoted the comparison between PLC-γ1_B vs SCR or PLC-γ1_A or in normoxic condition and # denoted the comparison between PLC-γ1_B vs SCR or PLC-γ1_A in hypoxic condition.

To examine if PLC-γ1 suppression has effects on chemotherapeutic drugs, therefore, we evaluated the effect of chemo-sensitivity of PLC-γ1 suppressed kasumi-1 cells. PLC-γ1 knockdown cells at day 4 after transduction were shown to induce significant cellular sensitivity to the genotoxic agents; mitoxantrone (p<0.05, p<0.001), amsacrine (p<0.01, p<0.001) and etoposide (p<0.05, p<0.01 and p<0.001) in kasumi-1 in a dose-dependent manner (Figure 5A–5C). Additionally, we evaluated the cell growth of PLC-γ1 knockdown cells under another stress condition, hypoxia. The cells were kept under hypoxia condition for 48h. Cell count analysis revealed a significant decreased in cell number in PLC-γ1-B construct compared to SCR in hypoxic condition (p<0.05, Figure 5D).

## DISCUSSION

AML is a life threatening malignancy in children. The t(8;21) (q22;q22) chromosomal translocation is one of the most frequent genetic alterations in AML. About 30% t(8;21) AML patients undergo relapse which indicates the need for improved therapeutic strategies [14–18]. Therefore, the development of new therapeutic strategies for t(8;21) AML is necessary. We identified PLC-γ1 as a potential target for t(8;21) AML from our peptide microarray profiling. PLC-γ1 is known to play a role in the progression of several carcinomas by activating PI3K/AKT/mTOR and MAPK pathways which regulate cell proliferation and survival [27–29, 31]. The knowledge about the role of PLC-γ1 in leukemia progression is very limited. Only one study showed that in CML, PLC-γ1 involved in leukemogenesis via a novel Akt-independent, PLC-γ1-driven mechanism of activation of mTOR/p70S6-K [30]. Therefore, it is important to investigate whether PLC-γ1 contributes in the regulation of t(8;21) AML.

In this study, we described a novel role of PLC-γ1 in t(8;21) AML. At first, we found higher peptide phosphorylation of PLC-γ1 at a tyrosine residue Y783 relative to NBM and CN-AML which was opposite to PLC-γ1 serine residue (S1248). PLC-γ1_Y783 is known to be a critical phosphorylation site for PLC-γ1 enzymatic activation whereas PLC-γ1_S1248 negatively regulates PLC-γ1 activity [23, 24]. The PLC-γ1 peptide phosphorylation was confirmed at protein levels both phosphorylation and total protein of PLC-γ1 in primary t(8;21) AML samples and kasumi-1 cells compared to other karyotypes and cell lines. Publicly available gene expression data of PLC-γ1 confirmed the higher expression of PLC-γ1 in t(8;21) AML. In addition, we also observed suppression of PLC-γ1 in AML1–ETO knock down kasumi-1 cells which indicated PLC-γ1 downregulation upon AML1-ETO suppression that might induce more kasumi-1 cell death. In previous studies, AML1-ETO was considered to be the most attractive target for the specific suppression of the t(8;21) translocation positive tumors although the effectiveness of this therapy remains unclear [7, 12]. So far no evidence has been published that PLC-γ1 could be a direct target for AML1-ETO. Based on the sequence matching analysis, we found that AML1-ETO consensus sequence is present in (−) 4081 base pairs upstream of transcription start site (TSS) which could be a regulatory region of PLC-γ1. Therefore, further study is needed to investigate in details.

Secondly, we investigated the shRNA-mediated inhibition of PLC-γ1 as currently available PLC-γ1 inhibitor U73122 is very unspecific [32]. PLC-γ1 knockdown in kasumi-1 cells resulted in repression of cell proliferation and more importantly, enhanced apoptosis which appeared to be the net results of the changes in the cell cycle. Immunoblot analysis confirmed the alternation of apoptosis and cell cycle regulation. Furthermore, we performed gene expression profiling to elucidate the phenotypic effects observed in response to shRNA mediated PLC-γ1 knockdown. Briefly, we observed up-regulation of apoptotic and DNA damage response genes together with the down regulation of cell growth, proliferation and differentiation genes, consistent with the observed phenotypic effects. Importantly, genes involve in Ca^2+^ signaling (CAMK2B and RYR1) were also downregulated in PLC-γ1 knockdown cells which are known to be altered via PLC-γ1 [33, 34]. Thus, the observed alternations of these genes in response to shRNA mediated PLC-γ1 knockdown contributes to the strong phenotypic effects found in our data.

At the end, we evaluated the effect of chemo-sensitivity of PLC-γ1 suppressed kasumi-1 cells. We found that PLC-γ1 knockdown cells were shown to have a higher cellular sensitivity to the genotoxic agent mitoxantrone, amsacrine and etoposide which indicated a possible future scope of synergistic effect of chemotherapeutics drugs together with PLC-γ1 inhibition although PLC-γ1 specific inhibitor needs to be developed at first. Similar approaches have been successfully used in different studies [34–37]. In addition, hypoxic stress showed less cell growth upon PLC-γ1 suppression.

In conclusion, we demonstrated higher PLC-γ1 phosphorylation and expression in t(8;21) AML. Moreover, our *in vitro* findings suggest an important role of PLC-γ1 in the survival of t(8;21) AML. Thus, PLC-γ1 may have important function in t(8;21) AML leukemogenesis. Therefore, these results emphasize the need for future investigation validating the role of PLC-γ1 as potential therapeutic targets for t(8;21) AML and it showed a possibility to use a combination therapy of anti AML1-ETO with anti PLC-γ1 for t(8;21) AML.

## MATERIALS AND METHODS

### AML patient samples and peptide microarray

Primary blood or bone marrow samples of newly diagnosed pediatric AML patients of t(8;21) AML (n=13), cytogenetically normal (CN-AML) (n=17) and bone marrow from healthy control (n=4) were collected after obtaining written informed consent in accordance with the declaration of Helsinki and the study was approved by the Medical Ethical Committee of the University Medical Center Groningen (UMCG). The associated patient characteristics of AML patients are described in Supplementary Table 1. Briefly, mononuclear cells were separated by lymphoprep density gradient (Nycomed, Oslo, Norway), and cryopreserved in liquid nitrogen until use. The cryopreserved leukemia cells were thawed rapidly at 37°C and diluted in a 6 ml volume of newborn calf serum, as described previously [20]. The remaining blast cell population contained >95% leukemia cells with PI staining, as shown in our previous study and is referred to hereafter as leukemia cells [20]. Previously, we used a high-throughput PepChipTM Kinomics microarray system (Pepscan, Lelystad, The Netherlands) to determine the peptide phosphorylation profiles of AML samples as described previously [20, 22]. This array contains 976 different kinase peptide substrates, each spotted as triplicates. The protein-derived peptide sequences contain phosphorylation sites that can be used as substrates for kinases active in the samples. The assay readout is the net sum of phosphorylation at each peptide, whether acted on by one kinase, or several different kinases.

### AML cell lines

OCI AML3, Kasumi-1, ME-1 and THP-1 cell lines are obtained from American Type Culture Collection (ATCC) (Manassas, VA, USA). Kasumi-1 is characterized by a t(8;21) chromosome translocation. This translocation juxtaposes the AML1 with ETO (or MTG8) gene, giving rise to the fusion gene AML1-ETO. All cell lines are cultured in RPMI-1640 medium supplemented with 1% penicillin/streptomycin and 10-20% fetal calf serum (Bodinco, Alkmaar, The Netherlands).

### shRNA-mediated knockdown

Lentiviral vector (pLKO.1) containing shRNA sequences targeting PLC-γ1 were obtained from Open Biosystems (Waltham, MA, USA) and genetically modified into a pLKO1-mCherry vector. Sequences are available upon request. Lentiviral particles were generated by co-transfection of the pLKO1-mCherry-shPLC-γ1 or scrambled vector (SCR) with packaging plasmid psPAX2 and the envelope plasmid pMD2.G into 293T cells using FuGENE HD transfection reagent (Roche, Woerden, The Netherlands) as described previously [38]. Kasumi-1 cells were incubated with lentiviral supernatants for two consecutive days after which stably transduced cells were expanded. The mCherry expression was measured to determine transduction efficiency by flow cytometry and the downregulation of PLC-γ1 was confirmed by RT-PCR and Western blot analysis. Subsequently, transduced cells were cultured and the rate cell proliferation was measured by absolute cell counting at every 48h using a Coulter Counter (Beckman Coulter, Fullerton, CA, USA) to assess cell growth in a time-dependent manner. The sequence of the AML1/MTG8 siRNA was previously described [39]. The lentiviral transgene plasmids pdc-SEW, AML1/MTG8- and shRNA-controls were cloned as described [40]. Lentiviral constructs encompassing the shRNAs encode GFP (green fluorescent protein) as a reporter gene. The preparation of recombinant lentiviral supernatants and lentiviral transductions were performed as described earlier [41]. Then transduced cells were cultured and cell pellets were made to confirm *AML1-ETO* knockdown and to check the PLC-γ1 protein expression at different days of transduction.

### Quantitative real-time PCR (qRT-PCR) and western blot analysis

The mRNA and protein expression of PLC-γ1 were assessed by quantitative real-time PCR (qRT-PCR) and western blot analysis respectively. Briefly, for qRT-PCR, total RNA was isolated (RNeasy mini kit, Qiagen, Hilden, Germany) and subsequent complementary DNA was synthesized from 1 μg RNA (First Strand cDNA Synthesis kit, Thermo Scientific, Waltham, MA, USA) according to manufacturer’s protocol. To check the knockdown PLC-γ1 mRNA expression was detected in triplicate using SYBR Green qRT-PCR (Applied Biosystems, Darmstadt, Germany) and normalized to RPL22 as a reference gene [42]. Relative mRNA expression level of the PLC-γ1 was calculated using the 2–ΔΔCT method relatively to SCR transduced cells.

For western blot, 1×10^6^ cells of newly diagnosed primary AML samples, cell lines, and PLC-γ1 transduced cells were lysed in laemmli sample buffer (Bio-Rad Laboratories, Veenendaal, The Netherlands). Proteins were separated by SDS-polyacrylamide gel electrophoresis and transported to nitrocellulose membranes as described previously [38]. First, the membranes were incubated overnight with primary antibodies for PLC-γ1, p-PLC-γ1, AML1, Bcl2, BAX, p-Chk2, p-p53 (Cell signalling, Danvers, MA, USA), t-p53 (BD Biosciences), t-Chk1, t-Chk2 (Santa Cruz) and GAPDH (Fitzgerald Inc.) followed by 1h incubation with HRP-conjugated secondary antibodies (DAKO Cytomation). Protein bands were visualized by chemiluminescence, using ChemiDoc MP system (Bio-rad laboratories) and normalized using GAPDH.

### Flow cytometry for apoptosis, cell proliferation, and cell cycle analysis

For apoptosis analysis upon PLC-γ1 knockdown, transduced cells were stained with Annexin V-FITC (Annexin-V-FLUOS Staining Kit, Roche) (1ml staining buffer, with the addition of 20μl Annexin V-FITC) as described previously [43]. For cell proliferation and cell cycle analysis, transduced cells were incubated with BrdU (5-bromo-20 -deoxyuridine) for 4 hours at 37°C after which the cells were fixed, denaturated, and stained with anti-BrdU Rabbit anti-Mouse FITC–conjugated secondary antibody (Dako cytomation) for 30 min at room temperature in the dark and then cells were stained with propidium iodide (PI) (Roche, Woerden, the Netherlands) for an additional 15 minutes in the dark at 37°C as described previously [43, 44]. All flow cytometry experiments were performed on a LSR-II flow cytometer (BD FACS DIVA software, BD bioscience, Breda, The Netherlands) and analyzed using FlowJo software (Tree Star Inc., Ashland, OR, USA). The cell cycle distribution was determined by flow cytometry for DNA content. Results were presented as a percentage of kasumi-1 cells (transduced with PLC-γ1 or with SCR) in each phase of cell cycle in relation to a total number of cells counted.

### Induction of hypoxia

For induction of hypoxia, *shPLC-γ1* and *shSCR* transduced kasumi-1 cells were kept in an air tight with a pouch (Gaspak, Becton Dickinson) without oxygen for 48h at 37°C as described previously [45]. The rate cell proliferation was measured by absolute cell counting after 48h using a Coulter Counter (Beckman Coulter, Fullerton, CA, USA) to assess cell growth under hypoxia and normoxia.

### Chemo-sensitivity and cell viability assay

Quantification of Kasumi-1 cell viability (SCR vs shPLC-γ1) was carried out using WST-1 assays with and without chemotherapeutic drugs as described previously [20]. These assays were performed in sextuple according to manufacturer’s protocol (Roche). Cells were seeded at a density of 0.1–1×10^5^ cells per 100 μl/well in a 96 wells plate in RPMI medium supplemented with 1% fetal calf serum with and without chemotherapeutic drugs (range of concentration), mitoxantrone (0.1-1 μM, Sandoz BV, The Netherlands), amsacrine (0.25-1 μg/mL, ProStrakan Pharma, The Netherlands) and etoposide (2.5-10 μM, Pharmachemie BV, The Netherlands). The mitochondrial activity of AML cell was measured after 48 h using a microplate reader at 450 nm (Benchmark; Bio-Rad Laboratories). Cell survival percentages (viability) are determined relative to non-treated cells.

### Gene expression and pathway analysis

Total RNA was isolated from transduced kasumi-1 cells of scrambled and *shPLC-γ1* with the RNeasy mini kit. Further processing including the quality control, RNA labeling, hybridization, and data extraction was performed at ServiceXS B.V. (Leiden, The Netherlands) using Illumina HumanHT-12 v4.0 Expression BeadChips as previously described [38]. Briefly, scans (Illumina iScan), image analysis and extraction of raw data (Illumina GenomeStudio v2011.1) were done by ServiceXS B.V. Then, we performed quantile normalization, log2-transformation, and group comparisons. Furthermore, a system biology pathway analysis tool, MetaCore^TM^ (GeneGo Inc., St Joseph, MI, USA), was used to explore the signaling pathways between scrambled and *shPLC-γ1* kasumi-1 cells.

### Statistical analysis

The comparison of patient characteristics between t(8;21) AML and CN-AML were assessed in SPSS (Statistical Package for the Social Sciences). Mann-Whitney U test (for continuous variables) and Pearson Chi-Square test (for categorical variables) were used to assess the difference between these two groups of patients. Other data were expressed as means+SEM unless described differently. Comparisons between groups were made using non-parametric tests (Mann–Whitney U) and the parametric paired and unpaired two-tailed t-test, a *P-value* less than 0.05 (alpha) was considered to denote statistically significant difference.

## SUPPLEMENTARY MATERIALS FIGURES AND TABLE



## Abbreviations

AML: Acute myeloid leukemia
NBM: Normal bone marrow
PLC-γ1: Phospholipase C-gamma 1
A-E or RUNX1-RUNX1T1: AML1-ETO
CN-AML: Cytogenetically normal AML
IP3: Inositol-1,4,5-triophosphate
DAG: 1,2-diacylglycerol
VEGF: Vascular endothelial growth factor
CML: Chronic myeloid leukemia
PKC: Protein kinase C
qRT-PCR: Quantitative real-time PCR
PI: Propidium iodide
SPSS: Statistical Package for the Social Sciences
