# Supplementary material for: Peptide microarray profiling identifies phospholipase C gamma 1 (PLC-γ1) as a potential target for t(8;21) AML
Source: Oncotarget. 2017 Jun 27;8(40):67344–54. doi: 10.18632/oncotarget.18631 (PMC5620177; doi:10.18632/oncotarget.18631)
Supplement: Supplementary file 1 [file oncotarget-08-67344-s001.pdf]

Peptide microarray profiling identifies phospholipase C gamma 1 (PLC-γ1) as a potential target for t(8;21) AML

SUPPLEMENTARY FIGURES AND TABLE

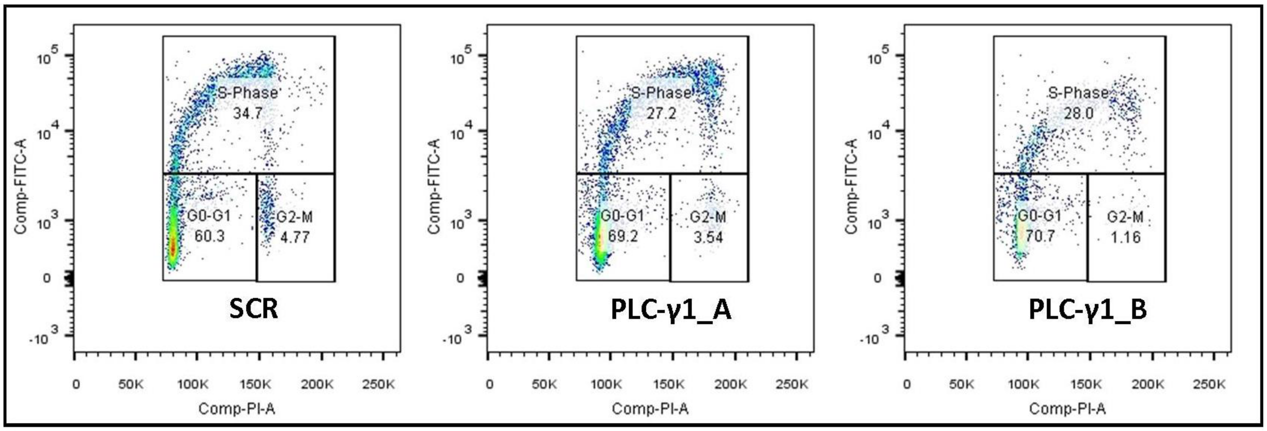

Supplementary Figure 1: Suppression of PLC-γ1 in kasumi-1 cells induces G0/G1 phase cell cycle arrest. The exact changes of cell cycle phases are depicted in the original dot plot from the FACS analysis. Cell cycle analysis of PLC-γ1 knockdown cell revealed a significant increase the percentage of cells in G0 /G1 phase and significantly decreased in S-phase and G2/M.

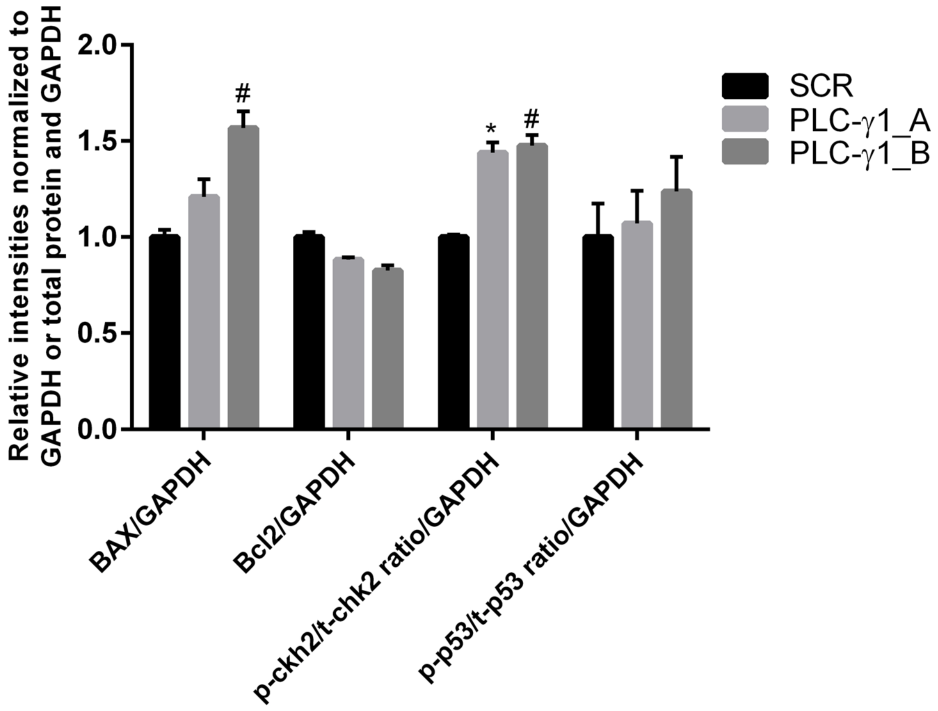

Supplementary Figure 2: The quantification of the western blot analysis of Figure 4C. The quantification of the western blot analysis is presented as a bar diagram. For BAX and Bcl2 protein levels were normalized against GAPDH. Phosphorylation of Chk2 and p53 proteins were normalized with their total protein levels and further with GAPDH. Significant differences between two individual groups were determined by using Student's t-test, $p<0.05$. * denoted the comparison between SCR vs PLC-γ1_A; # denoted the comparison between SCR vs PLC-γ1_B.

Supplementary Table 1: Patient characteristics

Characteristics	t(8;21) (n=13)	CN-AML (n=17)	*p-value*
**Age (year)**			
**Median**	10	11	*0.57*
**Range**	4-15	2-14	
**Gender (%) p=0.07**			
**Male**	11	9	*0.07*
**Female**	2	8	
**BM blast (median %)**	67	73.5	*0.14*
**PB blast (median %)**	58.5	76.5	*0.16*
**WBC, 10^9/L (median)**	73.6	25.3	*0.002***
**HGB, 10^9/L (median)**	5.05	5	*0.85*
**PLT, 10^7/L (median)**	37	43	*0.40*
**Relapse**	5/13	7/17	*0.77*
**Death**	5/13	6/17	*0.86*
